# Recovery and the use of postoperative physical therapy after total hip or knee replacement

**DOI:** 10.1186/s12891-022-05429-z

**Published:** 2022-07-13

**Authors:** L. Groot, D. A. J. M. Latijnhouwers, M. Reijman, S. H. M. Verdegaal, T. P. M. Vliet Vlieland, M. G. J. Gademan, on behalf of the Longitudinal Leiden Orthopaedics Outcomes of Osteoarthritis Study (LOAS) Group, R. G. H. H. Nelissen, H. M. J. van der Linden, B. L. Kaptein, P. J. Damen, H. H. Kaptijn, S. B. W. Vehmeijer, W. J. C. M. Marijnissen, R. Onstenk

**Affiliations:** 1 Department of Orthopedics and Sports Medicine, Erasmus MC, University Medical Center, Rotterdam, the Netherlands; 2 Department of Orthopaedics, Rehabilitation and Physical Therapy Leiden, Leiden University Medical Center, Leiden, the Netherlands; 3 Department of Orthopedics, Alrijne Hospital, Leiden and Leiderdorp, The Netherlands; 4 Department of Clinical Epidemiology, Leiden University Medical Center, Albinusdreef 2, 2300 RA Leiden, the Netherlands

**Keywords:** Physical therapy, Recovery, Hip, Knee, Arthroplasty, Osteoarthritis

## Abstract

**Background:**

Total hip or knee arthroplasties (THA/TKA) show favorable long-term effects, yet the recovery process may take weeks to months. Physical therapy (PT) following discharge from hospital is an effective intervention to enhance this recovery process. To investigate the relation between recovery and postoperative PT usage, including the presence of comorbidities, 6 months after THA/TKA.

**Methods:**

Multicenter, observational study in primary THA/TKA patients who completed preoperative and 6 months postoperative assessments. The assessments included questions on PT use (yes/no and duration; long term use defined as ≥ 12 weeks), comorbidities (musculoskeletal, non-musculoskeletal, sensory comorbidities and frequency of comorbidities). Recovery was assessed with the HOOS/KOOS on all 5 subdomains. Logistic regression with long term PT as outcome was performed adjusted for confounding including an interaction term (comorbidity*HOOS/KOOS-subdomain).

**Results:**

In total, 1289 THA and 1333 TKA patients were included, of whom 95% received postoperative PT, 56% and 67% received postoperative PT ≥ 12 weeks respectively. In both THA and TKA group, less improvement on all HOOS/KOOS domain scores was associated with ≥ 12 weeks of postoperative PT (range Odds Ratios 0.97–0.99). In the THA group the impact of recovery was smaller in patient with comorbidities as non- musculoskeletal comorbidities modified all associations between recovery and postoperative PT duration (Odds Ratios range 1.01–1.05). Musculoskeletal comorbidities modified the associations between Function-in-Daily-Living-and Sport-and-recreation recovery and postoperative PT. Sensory comorbidities only had an effect on Sport-and-recreation recovery and postoperative PT. Also the frequency of comorbidities modified the relation between Function-in-Daily-Living, pain and symptoms recovery and postoperative PT. In the TKA group comorbidity did not modify the associations.

**Conclusion:**

Worse recovery was associated with longer duration of postoperative PT suggesting that PT provision is in line with patients’ needs. The impact of physical recovery on the use of long-term postoperative PT was smaller in THA patients with comorbidities.

**Trial registration:**

Registered in the Dutch Trial Registry on March 13, 2012. TRIAL ID NTR3348; registration number: P12.047. https://www.trialregister.nl/trial/3197.

**Supplementary Information:**

The online version contains supplementary material available at 10.1186/s12891-022-05429-z.

## Introduction

Total hip or knee arthroplasties (THA/TKA) are effective procedures in patients with end stage hip or knee osteoarthritis (OA) [1, 2]. Overall, THA and TKA show favorable long-term effects, yet the recovery process may take weeks to months. Physical therapy (PT) following discharge from hospital is an effective intervention to enhance this recovery process [3–6]. Several guidelines recommend the use of PT to improve recovery after THA/TKA [7–9]. In Western Europe, post discharge PT after THA/TKA ranges from 35 to 99% [10–12]. A rise in use of postoperative PT is expected due to the foreseen increase in arthroplasty procedures [2, 13, 14].

Previous studies report variation in treatment modalities, the timing and/or the frequency and duration of post discharge PT indicating no clear consensus on its optimal dosage and timing [10, 15–19]. Also, studies identified determinants that affected use of inpatient versus outpatient PT treatment and its program intensity. It was found that patients referred to the less intensive outpatient PT were more often younger, had less comorbidities, a public insurance status and less functional disability, also referral depended on the treating hospital [10, 19–21] as compared to those having more intensive and/or inpatient PT. Understanding the relationships between these factors and the use of postoperative PT could provide better patient expectations, more patient specific advice and targeted postoperative PT care for patients after THA/TKA [22–25].

The patient’s physical recovery after THA/TKA, including the presence of comorbidities and its effect on the duration of postoperative PT, has not yet been extensively studied, while it is likely that patients with worse overall health status might have a greater need for PT. To our knowledge only the study of Smith et al., relying on data from the National Joint Registry for England, showed that worse functional outcomes at 12 months after THA/TKA were related to longer duration of PT. However, this relationship was not adjusted for patient characteristics [12].

Therefore, the aim of the present study was to examine the association between recovery and the duration of postoperative PT, taking into account individual patient characteristics and in particular the presence of comorbidities, as these can have a significant influence on the recovery after arthroplasty as well as PT [26, 27]. A better understanding of the relationships between these factors and the use of postoperative PT could provide new insights into the process of recovery after THA/TKA and targets for optimization of care.

## Methods

### Study design

This study was part of the ongoing multi-center, prospective cohort Longitudinal Leiden Orthopaedics Outcomes of Osteo-Arthritis study (LOAS) (Trial ID NTR3348) [28]. Ethical approval was obtained from the Medical Ethics Committee of the Leiden University Medical Center (Registration number P12.047). Since 2012 the LOAS includes consecutive patients scheduled for primary THA/TKA as a result of OA in seven participating hospitals: Leiden University Medical Center, Leiden; Alrijne Hospital, Leiden and Leiderdorp; Groene Hart Hospital, Gouda; Reinier de Graaf Hospital, Delft; Lange Land Hospital, Zoetermeer; Albert Schweitzer Hospital, Dordrecht; and Waterland Hospital, Purmerend. All participants provided informed consent form prior to start of the study in accordance with the Handbook for Good Clinical Research Practice of the World Health Organization and Declaration of Helsinki principles [29].

### Study population

Patients eligible for the LOAS were aged 18 years or older, physically and/or mentally able to complete questionnaires in Dutch and underwent primary THA/TKA for OA. The present study included a subset of patients from the LOAS who were included between June 2012 and June 2018, who filled out questions on the use of PT pre- and postoperatively and completed the questionnaires on hip/knee function and quality of life both preoperatively and 6 months after surgery. Patient who did not have postoperative PT were excluded from the analysis because they were not seen by a physical therapist after being discharged from the hospital.

### Assessments

#### Patient Characteristics

Patient characteristics were recorded preoperatively and included age (years), sex, living status (living alone / living with partner, children or other(s)), working status (having a paid job yes/no) and the Body Mass Index (BMI (kg/m2)).

In addition, information on self-reported pain, quality of life and comorbidities were gathered preoperatively. Hip and knee pain severity in rest and during activities in the past week were assessed by the Numeric Rating Scale (NRS) [30], with scores ranging between 0 (no pain) and 10 (worst pain imaginable). Regarding quality of life, the SF-12 was administered [31] from which the Physical and Mental Component Summary scales (PCS and MCS) were computed, with scores ranging between 0 (worst physical/mental health) and 100 (best physical/mental health). Self-reported comorbidities were gathered with a questionnaire developed by the Dutch Central Bureau of Statistics (CBS) [32, 33], in which the presence of comorbidities in the previous year was determined (yes/no). According to the paper of Peter et. al [27], comorbidities were classified into three domains: Musculoskeletal comorbidities (elbow, wrist, hand, or back pain; other rheumatic diseases), non‐musculoskeletal comorbidities (chronic lung, cardiac, or coronary disease; arteriosclerosis; hypertension; [consequences of] stroke; severe bowel disorder; diabetes mellitus; migraine; psoriasis; chronic eczema; cancer; urine incontinence) and sensory impairments (hearing or vision impairments; dizziness in combination with falling). Also the numbers of comorbidities were categorized into 4 groups: no comorbidities, 1–2 comorbidities, 3–4 comorbidities, and ≥ 5 comorbidities.

#### Physical therapy use

The use of preoperative PT was measured by one question: “Did you have contact with a PT before surgery for your hip or knee complaints in the past 6 months?” (yes/no). The use of postoperative PT was measured by three questions: “Did you receive physical therapy after surgery?” (yes/no); “What was the estimated duration of physical therapy?’’ (4 weeks, 6 weeks, 8 weeks or 12 weeks or more); and “What was the average frequency of physical therapy?” (Once a week, twice a week or three times per week or more). According to the Dutch guideline, long duration of PT was specified as 12 weeks of PT treatment or more [9].

#### Hip and knee related health status recovery and quality of life

The Hip disability and Osteoarthritis Outcome Score (HOOS) and the Knee injury and Osteoarthritis Outcome Score (KOOS) were used to assess hip and knee related symptoms (health status) preoperatively and 6 months after surgery [34, 35]. The questionnaires contain 40 and 42 items, respectively, categorized into five domains (function in daily living; pain; symptoms; sport and recreation; quality of life). All scores ranging from 0 (severe impairments) to 100 (no impairments). To determine the impact of recovery on the use of postoperative PT, all domains of the HOOS/KOOS were included. The extent of recovery was expressed as the absolute HOOS/KOOS domain scores at 6 months adjusted for the preoperative values. The distribution based Minimal Clinical Important Differences (MCID) for the HOOS/KOOS domains have been reported to be around 8–9 points [36].

### Statistical analysis

All analyses were performed for THA and TKA separately. Descriptive statistics (mean and standard deviation (SD), median with interquartile range or numbers and percentages) were used to present the patients’ preoperative characteristics, level of overall health status, pain and use of PT.

Baseline characteristics were presented according to postoperative PT (divided into none, < 12 weeks or ≥ 12 weeks), using One-way ANOVA, Student t tests, Mann–Whitney-U tests or Chi Square tests to test differences between these groups or combinations of these groups, where appropriate patients who did not make use of post-discharge PT were excluded from further analyses. To study the association between recovery and the duration of postoperative PT, the 6-month HOOS/KOOS separate domain absolute scores, as well as their change scores were presented for the total group of postoperative PT users and compared between the groups with short and long duration of postoperative PT by means of Paired t tests (results expressed as mean difference in change with the 95% confidence interval (CI)). In addition, to account for potential confounders, logistic regression analyses were performed, in which duration of postoperative PT (< or ≥ 12 weeks) was the dependent variable and the 6-month absolute scores of the HOOS/KOOS domain scores were each separately considered as independent variables. Comorbidities were included as possible effect modifier and the following potential confounders, which were based on previous literature: age, sex, BMI, baseline HOOS/KOOS scores, living status and mental health [22]. For each of the three different comorbidity domain groups (musculoskeletal, non- musculoskeletal and sensory impairments), we performed separate analyses as patients could have several comorbidities and therefore could be included in more than one comorbidity domain. In addition, we performed a separate analyses on the number of comorbidities (continuous variable) within a patient as possible effect modifier. All statistical analyses were performed on complete cases, two-sided with 95% Confidence Intervals (95%CI), using SPSS software (IBM Corp. Released 2016. IBM SPSS Statistics for Windows, Version 25.0. Armonk, NY: IBM Corp.).

## Results

### Study population

**Fig.1:**
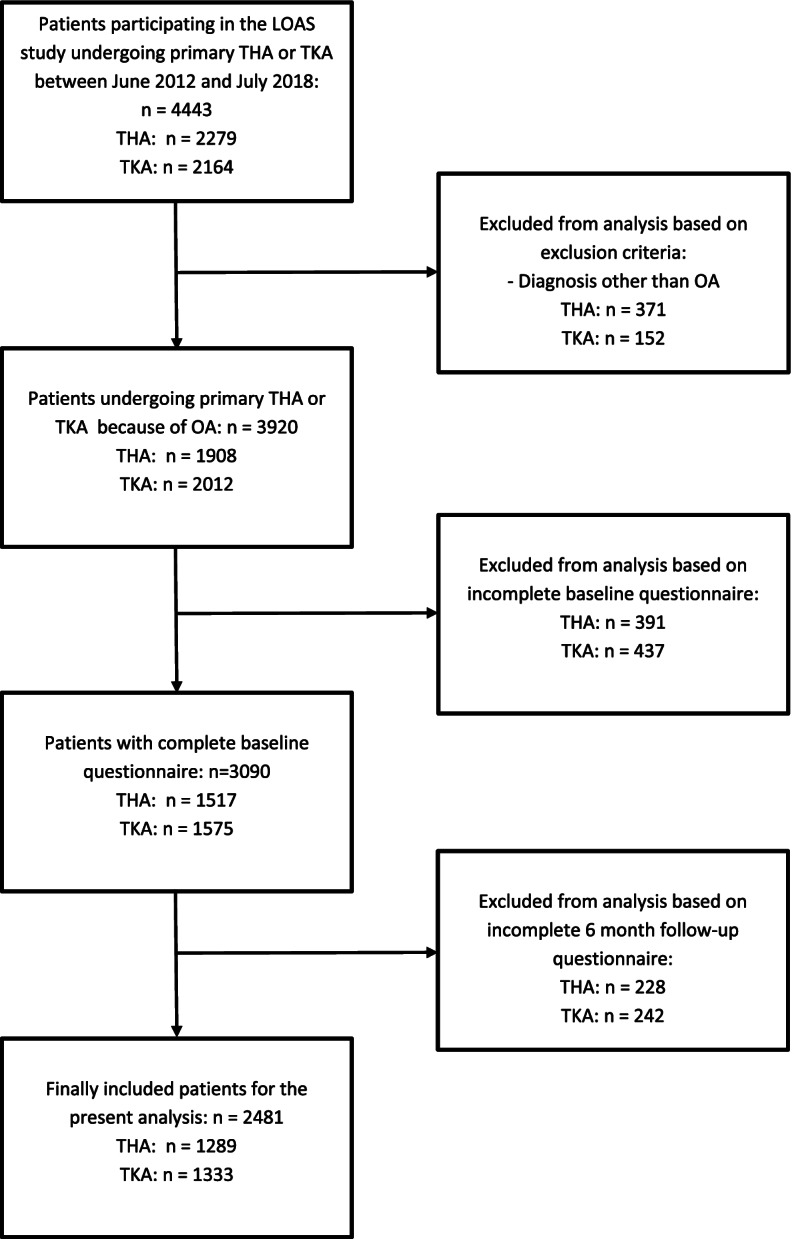
Flowchart of patients in our study on the use of physical therapy before and after total hip or knee arthroplasty. THA = total hip arthroplasty; TKA = total knee arthroplasty

The flow of participants in this study is presented in Fig 1. In total, 2279 and 2164 patients received a primary THA/TKA, respectively. Of these patients, 1289 THA and 1333 TKA patients were finally included for the present analysis. The included THA/TKA patients were on average somewhat younger, scored higher on the MCS and had a larger proportion of patients with ≥ 12 weeks of postoperative PT compared to the excluded patients (Supplementary table 1).

### Characteristics of THA and TKA patients

Table 1 shows the baseline characteristics of the patients. Both THA/TKA patients were on average 68 years old (THA SD 8.8; and TKA SD 8.4 years), and 60% and 63% of THA/TKA patients were female. Among patients younger than 67 years old, 55% and 53% of THA/TKA patients had a paid job before surgery. Overall, 39% of the THA patients had a musculoskeletal comorbidity, 62% had a non-musculoskeletal comorbidity and 5% had a sensory impairment. Of them 26.7% had a comorbidity in two domains and 2.3% in all 3 domains (Supplementary table 2). 42% of the TKA patients had a musculoskeletal comorbidity, 62% had a non-musculoskeletal comorbidity and 6% had a sensory impairment. Of them 27.3% had a comorbidity in two domains and 2.5% in all 3 comorbidity domains.

**Table 1:** Characteristics of the participating patients

	**THA**	**TKA**	**THA**				**TKA**			
	***n*** ** = 1289**	***n*** ** = 1333**	**No** **postoperative PT** n** = 110**	** < 12 weeks** **postoperative PT** ***n*** ** = 503**	** ≥ 12 weeks** **postoperative PT** ***n*** ** = 676**	**Difference between PT groups** **P Value**	**No** **postoperative PT** ***n*** ** = 31**	** < 12 weeks** **postoperative PT** ***n*** ** = 377**	** ≥ 12 weeks** **postoperative PT** ***n*** ** = 925**	**Difference between PT groups** ***P*** ** Value**
Sex, female; n (%)	771 (59.8)	844 (63.3)	63 (57.3)	279 (55.5)	429 (63.5)	0.018	7 (54.8)	218 (57.8)	609 (65.8)	0.015
Age, years	68.3 (8.8)	67.5 (8.4)	69.9 (8.1)	69.3 (8.5)	67.3 (9.1)	< 0.001	67.1 (9.6)	69.9 (7.9)	66.5 (8.4)	< 0.001
Body mass index; kg/m^2^	27.2 (4.2)	29.3 (4.6)	27.4 (4.9)	27.1 (4.1)	27.4 (4.2)	0.409	28.9 (5.4)	29.6 (4.7)	29.2 (4.5)	0.309
Living status; n (%)						0.015				0.015
- Alone	281 (21.8)	309 (23.2)	36 (32.7)	104 (20.7)	141 (20.9)		12 (38.7)	100 (26.5)	197 (21.3)	
- With others	1008 (78.2)	1024 (76.8)	74 (67.3)	399 (79.3)	535 (79.1)		19 (61.3)	277 (73.5)	728 (78.7)	
Working, yes; n (%)	306 (23.9)	327 (24.5)	23 (20.9)	103 (20.6)	180 (26.8)	0.036	5 (16.1)	57 (15.2)	265 (29.0)	< 0.001
Working < 67 years	280 (55.0)	303 (53.0)	21 (56.8)	91 (51.1)	168 (57.3)	0.412	5 (41.7)	49 (42.6)	249 (56.2)	0.024
Comorbidity*, yes, n (%)										
- Musculoskeletal	501 (38.9)	557 (41.8)	47 (42.7)	195 (38.8)	259 (38.3)	0.978	13 (41.9)	159 (42.2)	385 (41.6)	0.833
- Non‐musculoskeletal	795 (61.7)	821 (61.6)	71 (64.5)	291 (57.9)	433 (64.1)	0.381	20 (64.5)	238 (63.1)	536 (60.39)	0.170
- Sensory impairments	65 (5.0)	73 (5.5)	3 (2.7)	21 (4.2)	41 (6.1)	0.176	4 (12.9)	23 (6.1)	46 (5.0)	0.121
Comorbidity, count, n (%)						0.45				0.23
- 0	214 (16.6)	184 (13.8)	14 (12.7)	88 (17.5)	112 (16.6)		5 (16.1)	43 (11.4)	136 (14.7)	
- 1 or 2	738 (57.3)	727 (54.5)	74 (67.3)	294 (58.4)	370 (54.7)		16 (51.6)	207 (54.9)	504 (54.5)	
- 3 or 4	280 (21.7)	334 (25.1)	18 (16.4)	97 (19.3)	165 (24.4)		8 (25.8)	97 (25.7)	229 (24.8)	
- 5 or more	57 (4.4)	88 (6.6)	4 (3.6)	24 (4.8)	29 (4.3)		2 (6.5)	30 (8.0)	56 (6.1)	
Preoperative PT, n (%)	728 (56.5)	676 (50.7)	63 (57.3)	246 (50.9)	419 (64.3)	< 0.001	13 (41.9)	167 (47.7)	496 (55.6)	0.037
Postoperative PT; n (%)						0.433				0.028
- Primary care	1038 (89.6)	1171 (92.3)	-	437 (88.8)	601 (90.2)		-	331 (89.7)	840 (93.3)	
- Secondary care	120 (10.4)	98 (7.7)	-	55 (11.2)	65 (9.8)		-	38 (10.3)	60 (6.7)	
Frequency postoperative PT; n (%)			-			< 0.001				< 0.001
- 1 × per week	556 (47.5)	397 (30.6)	-	295 (59.4)	261 (38.8)		-	178 (47.5)	219 (23.7)	
- 2 × per week	572 (48.9)	853 (65.7)	-	178 (35.8)	394 (58.5)		-	190 (50.7)	663 (71.8)	
- ≥ 3 × per week	42 (3.6)	48 (3.7)	-	24 (4.8)	18 (2.7)		-	7 (1.9)	41 (4.4)	
HOOS or KOOS										
- Symptoms	40.9 (18.5)	49.8 (18.3)	42.6 (19.7)	42.3 (18.4)	39.6 (18.3)	0.030	50.7 (20.2)	50.9 (18.3)	49.3 (18.3)	0.345
- Pain	39.1 (18.5)	39.4 (17.4)	41.6 (22.7)	40.7 (18.5)	37.6 (17.7)	0.008	37.1 (16.5)	40.3 (16.5)	39.1 (17.8)	0.404
- ADL	41.6 (19.2)	45.9 (18.1)	43.1 (21.5)	43.4 (19.0)	39.9 (18.8)	0.007	44.8 (21.6)	46.8 (17.7)	45.6 (18.2)	0.544
- Sport	19.6 (18.9)	10.7 (14.2)	20.4 (18.7)	20.5 (19.1)	18.7 (18.8)	0.236	10.5 (16.5)	11.5 (14.4)	10.4 (14.1)	0.452
- Quality of life	29.5 (16.4)	26.5 (15.3)	29.6 (16.8)	31.3 (17.3)	28.2 (15.5)	0.007	25.3 (17.0)	27.6 (15.5)	26.1 (15.1)	0.266
NRS pain score;										
- During rest	4.6 (2.5)	4.7 (2.6)	4.0 (2.5)	4.7 (2.4)	4.8 (2.6)	0.014	5.8 (2.9)	4.3 (2.5)	4.8 (2.6)	0.035
- During activity	6.8 (2.2)	6.7 (2.5)	6.5 (2.3)	6.7 (2.3)	6.9 (2.1)	0.287	7.4 (2.5)	6.4 (2.7)	6.7 (2.4)	0.230
SF-12										
- MCS	53.7 (10.2)	54.9 (9.6)	53.4 (11.7)	54.1 (9.8)	53.4 (10.3)	0.434	53.6 (10.7)	55.1 (9.6)	54.8 (9.6)	0.705
- PCS	32.9 (6.2)	32.9 (6.1)	32.5 (6.5)	33.2 (6.3)	32.8 (6.0)	0.333	33.3 (6.1)	32.9 (6.1)	32.8 (6.1)	0.895
Duration of PT; n (%)										
- No PT	110 (9.1)	31 (2.2)								
- 4 weeks	126 (10.4)	75 (5.3)								
- 6 weeks	160 (13.2)	142 (10.1)								
- 8 weeks	217 (17.9)	160 (11.3)								
- > 12 week	676 (55.9)	925 (65.5)								

Data is presented as mean and standard deviation between parentheses or reported otherwise

HOOS = Hip disability and Osteoarthritis Outcome Score; KOOS = Knee injury and Osteoarthritis Outcome Score; NRS = Numeric Rating Scale; SF-12 = Short Form-12; THA = total hip arthroplasty; TKA = total knee arthroplasty; PT = Physical therapy

^*^The 3 comorbidity groups are not mutually exclusive

### Use of preoperative and postoperative PT

Table 1 shows that 728 (57%) THA patients and 676 (51%) of the TKA patients received preoperative PT. After surgery 91% of THA patients and 98% of TKA received PT. The large majority used primary care PT and 676 (55.9%) THA patients and 925 (65.5%) TKA patients used it for 12 weeks or more, whereas 49% of THA patients and 66% of TKA patients reported an average frequency of PT of twice a week.

Regarding the comparison of the characteristics of patients who had no postoperative PT or who had < 12 weeks PT versus those who had ≥ 12 weeks postoperative PT, it appeared that in both THA/TKA, patients with ≥ 12 weeks postoperative PT were more often female, younger and employed. Also, they used preoperative PT more often and received ≥ 2 sessions of postoperative PT treatments per week than patients who received PT < 12 weeks. Additionally, the THA group with ≥ 12 weeks postoperative PT scored lower on all HOOS subdomains apart from subdomain sport at baseline compared to the THA group with PT < 12 weeks. The TKA group with ≥ 12 weeks postoperative PT received PT in primary care more often. No differences on KOOS subdomains were found between both groups.

### Physical recovery and postoperative PT

Table 2 shows the crude absolute HOOS/KOOS domain scores at 6 months as well as their changes compared to the preoperative scores for patients who received < 12 weeks postoperative PT and those who received ≥ 12 weeks postoperative PT. We found that the group with ≥ 12 weeks postoperative PT had smaller improvements in HOOS/KOOS domain scores at 6 months. All differences between the groups were clinically relevant.

**Table 2:** Physical recovery and postoperative physical therapy after total hip or knee arthroplasty surgery

	**THA**					**TKA**				
	**postoperative PT** ** < 12 weeks** ***n*** ** = 503**		**postoperative PT** ** ≥ 12 weeks** ***n*** ** = 676**			**postoperative PT** ** < 12 weeks** ***n*** ** = 377**		**postoperative PT** ** ≥ 12 weeks** ***n*** ** = 925**		
**HOOS/ KOOS domain score**	**6 months** **mean (SD)**	**Change score 6 months** **(95% CI)**	**6 months** **mean (SD)**	**Change score 6 months (95% CI of mean difference)**	**Mean difference in change score between groups (95% CI of mean difference)**	**6 months** **Mean (SD)**	**Change score 6 months (95% CI)**	**6 months** **Mean (SD)**	**Change score 6 months (95% CI of mean difference)**	**Mean difference in change score between groups (95% CI of mean difference)**
Symptoms	82.7 (16.9)	40.8 (42.9 – 38.8)	73.9 (21.2)	34.5 (36.3 – 32.6)	8.8 (6.6 – 11.0)	77.5 (15.0)	25.9 (28.1 – 23.6)	69.1 (17.6)	19.7 (21.2 – 18.1)	9.5 (7.4 – 11.6)
Pain	90.6 (12.8)	50.4 (52.2 – 48.5)	83.5 (19.3)	45.9 (47.7 – 44.1)	7.1 (5.3 – 9.0)	86.6 (16.2)	46.0 (48.3 – 43.7)	77.1 (20.2)	38.2 (39.8 – 36.6)	8.4 (6.5 – 10.3)
Activities of daily living	87.3 (14.2)	44.5 (46.3 – 42.6)	79.3 (19.4)	39.7 (41.5 – 43.7)	8.0 (6.0 – 9.9)	84.4 (16.8)	37.1 (39.3 – 34.9)	77.4 (19.1)	31.9 (33.4 – 30.5)	7.0 (4.9 – 9.1)
Sport	69.0 (25.1)	48.4 (51.0 – 45.8)	59.0 (28.2)	40.5 (42.7 – 38.2)	10.0 (6.9 – 13.1)	47.7 (28.3)	36.5 (39.5 – 33.5)	35.4 (26.3)	25.2 (27.0 – 23.5)	12.3 (8.9 – 15.8)
Quality of life	79.9 (19.5)	48.8 (51.0 –46.6)	68.9 (22.5)	40.7 (42.7 – 38.8)	11.0 (8.5 – 13.4)	68.3 (20.9)	40.3 (42.7 – 37.9)	58.9 (21.2)	32.9 (34.4 – 31.3)	9.4 (6.9 – 12.0)

HOOS = Hip disability and Osteoarthritis Outcome Score (range 0–100); KOOS = Knee disability and Osteoarthritis Outcome Score (range 0–100); PT = physical therapy; THA = total hip arthroplasty; TKA = total knee arthroplasty

### Associations of recovery, comorbidities and duration of postoperative PT

Tables 3 and 4 show the adjusted Odds Ratios of the associations between recovery on all subdomains of the HOOS/KOOS and the duration of postoperative PT including the influence of comorbidities within these associations. For both THA/TKA we found that better recovery at 6 months was associated with shorter postoperative PT on all subdomains of the HOOS/KOOS (Tables 3 and 4, model 1).

**Table 3:** Association of recovery and duration of postoperative physical therapy in total hip arthroplasty patients 6 months after surgery

	**Model 1** **Multivariable** **OR [ 95% CI]**	**Model 1** **+ Musculoskeletal comorbidities** **OR [ 95% CI]**	**Model 1** **+ Non-musculoskeletal comorbidities** **OR [ 95% CI]**	**Model 1** **+ Sensory Impairments** **OR [ 95% CI]**	**Model 1** **+ frequency comorbidities** **OR [ 95% CI]**
HOOS ADL	0.97 [0.96 – 0.98]	0.96 [0.95 – 0.98]	0.95 [0.93 – 0.97]	0.97 [0.96 – 0.98]	0.96 [0.95 – 0.98]
Comorbidity	NA	0.15 [0.03 – 0.87]	0.71 [0.01 – 0.59]	0.23 [0.03 – 2.20]	0.70 [0.99 – 1.01]
ADL x comorbidity	NA	1.02 [1.00 – 1.04]	1.03 [1.01 – 1.06]	1.02 [0.99 – 1.05]	1.01 [1.00 – 1.01]
HOOS Pain	0.97 [0.97 – 0.98]	0.97 [0.96 – 0.99]	0.94 [0.91 – 0.96]	0.97 [0.96 – 0.98]	0.96 [0.95 – 0.97]
Comorbidity	NA	0.62 [0.10 – 3.75]	0.01 [0.001 – 0.17]	0.23 [0.02 – 2.71]	0.58 [0.38 – 0.88]
Pain x comorbidity	NA	1.01 [0.99 – 1.03]	1.05 [1.02 – 1.08]	1.02 [0.99 – 1.05]	1.01 [1.00 – 1.01]
HOOS Symptoms	0.98 [0.97—0.98]	0.97 [0.96 – 0.99]	0.96 [0.94 – 0.97]	0.98 [0.97 – 0.98]	0.97 [0.96 – 0.98]
Comorbidity	NA	0.39 [0.10 – 1.45]	0.19 [0.04 – 0.82]	0.52 [0.08 – 3.34]	0.72 [0.53 – 0.98]
Symptoms x comorbidity	NA	1.01 [0.96 – 1.03]	1.03 [1.0 – 1.04]	1.01 [0.99 – 1.04]	1.01 [1.00 – 1.01]
HOOS Sport and Recreation	0.99 [0.98 – 0.99]	0.98 [0.97 – 0.99]	0.98 [0.97 – 0.99]	0.99 [0.98 – 0.99]	0.98 [0.98 – 0.99]
Comorbidity	NA	0.39 [0.17 – 0.85]	0.74 [0.32 – 1.67]	0.52 [0.16 – 1.67]	0.90 [0.74 – 1.09]
Sport and Recreation x comorbidity	NA	1.02 [1.00 – 1.03]	1.01 [1.00 – 1.02]	1.02 [1.00 – 1.04]	1.00 [0.99 – 1.01]
HOOS Quality of life	0.98 [0.97 – 0.98]	0.97 [0.96 – 0.99]	0.97 [0.95 – 0.98]	0.98 [0.97 – 0.98]	0.96 [0.96 – 0.98]
Comorbidity	NA	0.64 [0.20 – 1.99]	0.99 [0.99 – 1.00]	0.51 [0.09 – 3.06]	0.73 [0.56 – 0.96]
Quality of life x comorbidity	NA	1.01 [0.99 – 1.02]	1.02 [1.00 – 1.03]	1.01 [0.99 – 1.04]	1.01 [1.00 – 1.01]

ADL = Activities of daily living; HOOS = Hip disability and Osteoarthritis Outcome Score; THA = total hip arthroplasty

Model 1: long duration of PT = bo + b1x (HOOS score) adjusted for BMI, age, sex, living status, and Short Form-12 Mental Component Summary scale and HOOS score baseline

In the other models comorbidities (musculoskeletal, non-musculoskeletal or sensory impairment and the frequency of comorbidities) and their interaction with recovery were also taken into account

**Table 4:** Association of recovery and duration of postoperative physical therapy in total knee arthroplasty patients 6 months after surgery

	**Model 1** **Multivariable** **OR [ 95% CI]**	**Model 1** **+ Musculoskeletal comorbidities** **OR [ 95% CI]**	**Model 1** **+ Non-musculoskeletal comorbidities** **OR [ 95% CI]**	**Model 1** **+ Sensory Impairments** **OR [ 95% CI]**	**Model 1** **+ frequency comorbidities** **OR [ 95% CI]**
KOOS ADL	0.97 [0.97 – 0.98]	0.99 [0.98 – 1.00]	0.98 [0.96 – 1.00]	0.97 [0.96 – 0.98]	0.97 [0.96 – 0.99]
Comorbidity	NA	5.78 [1.21 – 27.67]	1.64 [0.24 – 11.04]	1.41 [0.12 – 17.14]	0.94 [0.64 – 1.41]
ADL x comorbidity	NA	0.98 [0.96 – 1.00]	0.99 [0.97 – 1.01]	0.99 [0.96 – 1.03]	1.00 [1.00 – 1.01]
KOOS Pain	0.97 [0.96 – 0.98]	0.99 [0.97 – 1.00]	0.98 [0.97 – 1.00]	0.97 [0.96 – 0.98]	0.98 [0.96 – 0.99]
Comorbidity	NA	6.17 [1.23 – 30.92]	3.95 [0.66 – 23.60]	0.83 [0.06 – 2.32]	1.13 [0.72 – 1.76]
Pain x comorbidity	NA	0.98 [0.96 – 1.00]	0.98 [0.96 – 1.00]	1.00 [0.97 – 1.03]	1.00 [0.99 – 1.00]
KOOS Symptoms	0.97 [0.96 – 0.98]	0.98 [0.97 – 1.00]	0.97 [0.95 – 0.99]	0.97 [0.96 – 0.98]	0.97 [0.96 – 0.99]
Comorbidity	NA	3.55 [0.76 – 16.60]	0.93 [0.18 – 4.91]	5.56 [0.24 – 131.75]	1.04 [0.70 – 1.55]
Symptoms x comorbidity	NA	0.99 [0.97 – 1.01]	1.00 [0.98 – 1.02]	0.97 [0.93 – 1.02]	1.00 [0.99 – 1.00]
KOOS Sport and Recreation	0.98 [0.98 – 0.99]	0.99 [0.98 – 0.99]	0.98 [0.97 – 0.99]	0.98 [0.98 – 0.99]	0.98 [0.97 – 0.99]
Comorbidity	NA	0.98 [0.53 – 1.82]	0.92 [0.47 – 1.78]	1.24 [0.45 – 3.42]	0.93 [0.79 – 1.08]
Sport and Recreation x comorbidity	NA	1.00 [0.99 – 1.01]	1.00 [0.99 – 1.01]	0.98 [0.96 – 1.01]	1.00 [1.00 – 1.00]
KOOS Quality of life	0.98 [0.97 – 0.98]	0.99 [0.98 – 1.00]	0.98 [0.97 – 0.99]	0.98 [0.97 – 0.99]	0.98 [0.97 – 0.99]
Comorbidity	NA	2.27 [0.82 – 6.29]	0.90 [0.29 – 2.80]	1.05 [0.23 – 4.76]	1.00 [0.78 – 1.30]
Quality of life x comorbidity	NA	0.99 [0.97 – 1.00]	1.00 [0.98 – 1.02]	1.00 [0.97 – 1.02]	1.00 [1.00 – 1.00]

ADL = Activities of daily living; KOOS = Knee disability and Osteoarthritis Outcome Score; TKA = total knee arthroplasty

Model 1: long duration of PT = bo + b1x (KOOS score) adjusted for BMI, age, sex, living status, Short Form-12 Mental Component Summary scale and KOOS score baseline

In the other models comorbidities (musculoskeletal, non-musculoskeletal or sensory impairment and the frequency of comorbidities) and their interaction with recovery were also taken into account

In THA patients, the existence of non-musculoskeletal comorbidities modified the associations between recovery and the duration of postoperative PT (Table 3). In patients with a non-musculoskeletal comorbidity, the impact of physical recovery on the risk of long term PT was smaller than in the patients without musculoskeletal comorbidity (Supplementary figure 1-A). Hence in patients with a low HOOS subscore the probability of > 12 weeks of PT therapy was lower in patients with a non-musculoskeletal as compared to patients without a non-musculoskeletal comorbidity. However, in patients with high HOOS subscores this probability was higher in patients with a non-musculoskeletal comorbidity than in patients without such a comorbidity. Table 5 showed hypothetical examples of combined data within different HOOS / KOOS function scores to clarify our results. Moreover, the presence of a musculoskeletal comorbidity was identified as an effect modifier with regard to Activities of daily living (ADL) and Sport and recreation recovery and postoperative PT. Sensory comorbidities only had an effect on Sport and recreation recovery and postoperative PT. Also the number of comorbidities within a patient was identified as effect modifier in the HOOS subdomains ADL, pain and symptoms meaning the impact on recovery was lower in patients with more comorbidities. In TKA patients (Table 4) the existence of comorbidities as well as their frequency did not modify the associations between recovery and the duration of postoperative PT (Table 4).

**Table 5:** Hypothetical examples of combined data within different Hip disability and Osteoarthritis Outcome Score / Knee disability and Osteoarthritis Outcome Score function scores

Arthroplasty	HOOS / KOOS ADL score 6 months after surgery	Comorbidity	Risk on longer duration of PT after THA/TKA surgery
**Scenario: A female patient of 67 year, a BMI of 29, living with a spouse, HOOS ADL baseline score of 40 and a MCS of 54**			
**THA**			
1	75	Without musculoskeletal comorbidity	66%
2	75	With musculoskeletal Comorbidity	72%
3	75	Without non- musculoskeletal Comorbidity	70%
4	75	With non- musculoskeletal Comorbidity	57%
5	75	Without sensory impairment	68%
6	75	With sensory impairment	71%
7	89	Without musculoskeletal comorbidity	57%
8	89	With musculoskeletal Comorbidity	62%
9	89	Without non- musculoskeletal Comorbidity	60%
10	89	With non- musculoskeletal Comorbidity	50%
11	89	Without sensory impairment	58%
12	89	With sensory impairment	71%
13	95	Without musculoskeletal comorbidity	52%
14	95	With musculoskeletal Comorbidity	57%
15	95	Without non- musculoskeletal Comorbidity	55%
16	95	With non- Musculoskeletal Comorbidity	48%
17	95	Without sensory impairment	53%
18	95	With sensory impairment	71%
**Scenario: A female patient of 67 year, a BMI of 29, living with a spouse, KOOS ADL baseline score of 45 and a MCS of 55**			
**TKA**			
1	69	Without musculoskeletal comorbidity	82%
2	69	With musculoskeletal Comorbidity	72%
3	69	Without non- musculoskeletal comorbidity	80%
4	69	With non- musculoskeletal comorbidity	77%
5	69	Without sensory impairment	80%
6	69	With sensory impairment	78%
7	85	Without musculoskeletal comorbidity	73%
8	85	With musculoskeletal comorbidity	68%
9	85	Without non- musculoskeletal comorbidity	71%
10	85	With non- musculoskeletal comorbidity	70%
11	85	Without sensory impairment	72%
12	85	With sensory impairment	70%
13	94	Without musculoskeletal comorbidity	67%
14	94	With musculoskeletal Comorbidity	66%
15	94	Without non- musculoskeletal comorbidity	65%
16	94	With non- musculoskeletal comorbidity	66%
17	94	Without sensory impairment	67%
18	94	With sensory impairment	66%

BMI= body mass index; ADL = Activities of daily living; HOOS = Hip disability and Osteoarthritis Outcome Score; KOOS = Knee disability and Osteoarthritis Outcome Score; THA = total hip arthroplasty TKA = total knee arthroplasty; MCS = Mental Component Summary scale of the Short Form-12

Scenarios are based on the 25^th^, 50^th^ and 75th percentile of the HOOS/KOOS score 6 months after surgery

## Discussion

We evaluated whether the patient’s recovery 6 months after THA/TKA, including the presence of comorbidities, was related to the duration of postoperative PT. Approximately 95% of the study population received postoperative PT in a primary setting, where 49% of THA patients and 66% of TKA patients reported an average frequency of PT of twice a week. In about 56% of the THA and 66% of the TKA patients the average duration of postoperative PT was 12 weeks or more. Worse recovery at 6 months was associated with a longer duration of postoperative PT. The association between physical recovery and the use of long-term postoperative PT was weaker in THA patients with comorbidities than in patients without such a comorbidity. In TKA patients, comorbidities did not modify the association between recovery and postoperative PT duration.

Compared to Peter et al. [10], the frequency and duration of postoperative PT was slightly lower, but it was still substantially high compared to other Western countries [10–12]. Our findings regarding the patient’s recovery and duration of postoperative PT are in line with the study of Smith et al. [12]. They found that THA/TKA patients who received more than 10 postoperative PT sessions reported more complaints on average one year after surgery than patients who had less PT sessions. However, in the study of Smith et al., no adjustments were made and comorbidities were not taken into account. Nevertheless, the finding of both previous as well as the current study suggest that patients who were likely to have a greater need of PT due to insufficient recovery, indeed received it over a prolonged period. Hence, although currently clear evidence-based postoperative PT treatment protocols are absent, physical therapists seem to be able to adapt the PT treatment duration to the individual patient needs.

In 85% of our population at least one comorbidity was present, which is similar to previously published prevalence’s in OA patients, which ranged from 68%-85% [37–40]. In THA patients, the presence of musculoskeletal, non-musculoskeletal or sensory impairments and the frequency of comorbidities modified the association between recovery and postoperative PT in at least one of the HOOS domains. In THA patients with such or more comorbidities the effect of recovery on the use of long-term postoperative PT was smaller than patients without such or with less comorbidities. This may be explained by the impact a comorbidity has on the postoperative PT treatment. Hence, the presence of comorbidities may require a more tailored PT treatment approach, in which the physical complaints resulting from the comorbidity can have a more prominent role and the recovery of the THA surgery may be less leading [41]. For example, a patient with heart failure may not be able to perform strenuous exercise, thereby some complaints possibly cannot be improved as much as when these exercises could be performed. Moreover, for these patients improvement of the aerobic capacity may be an additional goal. As such, the PT goals will differ from patients without such a comorbidity. In TKA patients, the presence of comorbidities had no moderating effect on the effect of recovery and long-term PT. A possible explanation could be that recovery after TKA is more complex and takes a longer period of time than after THA [42], as such the role of a comorbidity on the PT treatment may be less pronounced.

Nonetheless, protocols could be important in the delivery of the most optimal composition, dosage and mode of PT treatment [43]. Hence, the rising number of arthroplasty surgeries and their accompanying strain on the health care systems and society as a whole warrant for a very efficient postoperative PT approach. Here there may be a role for a multidisciplinary consultation between health care providers (general practitioners, orthopedic surgeons, physical therapists), thereby improving communication, and increasing knowledge and agreement on rehabilitation treatment. Additionally, monitoring the recovery of a patient by a healthcare provider appears to be beneficial for better outcomes [44]. Future studies should focus on the possibilities to improve or accelerate recovery using PT, thereby decreasing the duration of PT for THA/TKA patients. The PT has an excellent position to monitor the recovery process and identify patients with insufficient improvement due to the fact that the PT sees a patient twice a week on average in the first weeks after surgery, as opposed to a regular consultation by the orthopedic surgeon which is usually scheduled once in this period.

A strength of this study is the large regional, multicenter prospective cohort study design, in which we obtained both preoperative and postoperative assessments. Secondly, we used validated surveys to obtain recovery scores after surgery. However, this could also be a possible limitation, as we asked patients at six months about the duration of PT, which could have induced some recall bias. Examination of medical records could have provided more reliable and additional information, such as the content of the PT treatment. However, this method could not be used in the framework of this study. A second limitation is that we did not have information on the severity of the different comorbidities. Another possible limitation is the inability to account for all potential confounders, as such residual confounding may still be an issue. Lastly, all patients were included in the Netherlands, therefore the results may not be one to one generalizable to other countries with different health insurance systems. Nevertheless, the sociodemographic and clinical characteristics of the participants were quite similar to those of persons who underwent THA/TKA in other observational studies [16, 27, 40, 45, 46].

## Conclusion

In conclusion, less recovery was associated with longer duration of postoperative PT use after THA/TKA. The impact of physical recovery on the use of long-term postoperative PT was smaller in THA patients with comorbidities, but not in TKA patients. Patients with longer postoperative PT use showed worse recovery than patients with a shorter duration of PT use, suggesting that PT provision is in line with patients’ needs.

## Supplementary Information

**Additional file 1.**

## Data Availability

Data is owned by a third party. The data underlying this article were provided by the LOAS study group by permission. The data will be shared on reasonable request to the corresponding author, with permission of the LOAS study group.

## Abbreviations

LOAS: Leiden Orthopaedics Outcomes of Osteoarthritis Study
PT: Physical therapy
THA: Total hip arthroplasty
TKA: Total knee arthroplasty
HOOS: The Hip disability and Osteoarthritis Outcome Score
KOOS: Knee injury and Osteoarthritis Outcome Score
OA: Osteoarthritis
BMI: Body Mass Index
NRS: Numeric Rating Scale
SF-12: Short Form-12
PCS: Physical Component Summary scale
MCS: Mental Component Summary scale
CBS: Dutch Central Bureau of Statistics
MCID: Minimal Clinical Important Differences
SD: Standard deviation
CI: Confidence interval
ADL: Activities of daily living
