# Supplementary material for: Recovery and the use of postoperative physical therapy after total hip or knee replacement
Source: BMC Musculoskelet Disord. 2022 Jul 13;23:666. doi: 10.1186/s12891-022-05429-z (PMC9277921; doi:10.1186/s12891-022-05429-z)
Supplement: Supplementary file 1 — Additional file 1. [file 12891_2022_5429_MOESM1_ESM.docx]

**SUPPLEMENTARY TABLE 1.** Baseline patients characteristics total hip and knee arthroplasty patients stratified for inclusion

|  | THA | | | TKA | | |
| --- | --- | --- | --- | --- | --- | --- |
|  | **Study population**  **n =1289** | **Excluded population**  **n =619** | **p-values** | **Study population**  **n =1333** | **Excluded**  **n = 679** | **p-values** |
| Sex, female; n (%) | 771 (59.8) | 423 (68.3) | 0.002 | 884 (63.3) | 454 (66.9) | 0.116 |
| Age, years | 68.3 (8.8) | 69.5 (9.9) | <0.001 | 67.5 (8.4) | 68.7 (8.8) | 0.003 |
| Body mass index; kg/m^2^ | 27.2 (4.2) | 27.4 (4.3) | <0.001 | 29.3 (4.6) | 29.8 (5.1) | 0.041 |
| Living status; n (%)  -Alone  -With others | 281 (21.8)  1008 (78.2) | 135 (21.8)  484 (78.2) | 0.001 | 309 (23.2)  1024 (76.8) | 116 (17.1)  563 (82.9) | 0.859 |
| Working, yes; n (%) | 306 (23.9) | 127 (20.5) | 0.246 | 327 (24.5) | 139 (20.5) | 0.119 |
| HOOS/KOOS   - Symptoms - Pain - ADL - Sport - Quality of life | 40.9 (18.5)  39.1 (18.5)  41.6 (19.2)  19.6 (18.9)  29.5 (16.4) | 39.9 (19.2)  37.0 (19.2)  38.9 (19.5)  18.2 (18.5)  27.2 (17.3) | 0.325  0.028  0.010  0.180  0.006 | 49.8 (18.3)  39.4 (17.4)  45.9 (18.1)  10.7 (14.2)  26.5 (15.3) | 49.1 (17.3)  38.0 (18.0)  43.9 (20.0)  11.5 (15.6)  26.3 (16.0) | 0.503  0.120  0.045  0.293  0.730 |
| NRS pain score;   - During rest - During activity | 4.6 (2.5)  6.8 (2.2) | 5.0 (2.6)  7.0 (2.1) | 0.030  0.055 | 4.7 (2.6)  6.7 (2.5) | 5.3 (2.5)  6.9 (2.2) | 0.001  0.142 |
| SF-12   - MCS - PCS | 53.7 (10.2)  32.9 (6.2) | 52.0 (11.3)  33.2 (6.3) | <0.001  0.380 | 54.9 (9.6)  32.9 (6.1) | 53.1 (11.2)  33.6 (7.1) | 0.003  0.008 |
| Duration of PT 6 months after surgery; n (%)   - No PT - <12 weeks - ≥12 weeks | 110 (8.5)  503 (39.0)  676 (52.4) | 390 (63.0)  98 (15.8)  131 (21.2) | <0.001 | 31 (2.3)  377 (28.3)  925 (69.4) | 367 (54.1)  110 (16.2)  202 (29.7) | <0.001 |

Legend to supplementary Table 1. Data is presented as mean and standard deviation between parentheses or reported otherwise.

HOOS= Hip disability and Osteoarthritis Outcome Score; KOOS= Knee injury and Osteoarthritis Outcome Score; NRS= Numeric Rating Scale; SF-12 = Short Form-12; THA=total hip arthroplasty; TKA= total knee arthroplasty; PT= Physical therapy

**SUPPLEMENTARY TABLE 2.** Different comorbidity groups of the total study population stratified for total hip and knee arthroplasty patients

| Comorbidity group | THA  N= 1289 | TKA  N= 1333 |
| --- | --- | --- |
| Musculoskeletal, yes, n (%) | 501 (38.9) | 557 (41.8) |
| Non-musculoskeletal, yes, n (%) | 795 (61.7) | 821 (61.6) |
| Sensory impairment, yes, n (%) | 65 (5.0) | 73 (5.5) |
| Musculoskeletal + non-musculoskeletal, yes, n (%) | 310 (24.1) | 328 (24.6) |
| Musculoskeletal + sensory impairment, yes, n (%) | 13 (1.0) | 19 (1.4) |
| Non-musculoskeletal + sensory impairment, yes, n (%) | 20 (1.6) | 17 (1.3) |
| Musculoskeletal + non-musculoskeletal + sensory impairment, yes, n (%) | 30 (2.3) | 33 (2.5) |

Legend to supplementary table 2; THA=total hip arthroplasty; TKA= total knee arthroplasty

All groups are mutually exclusive


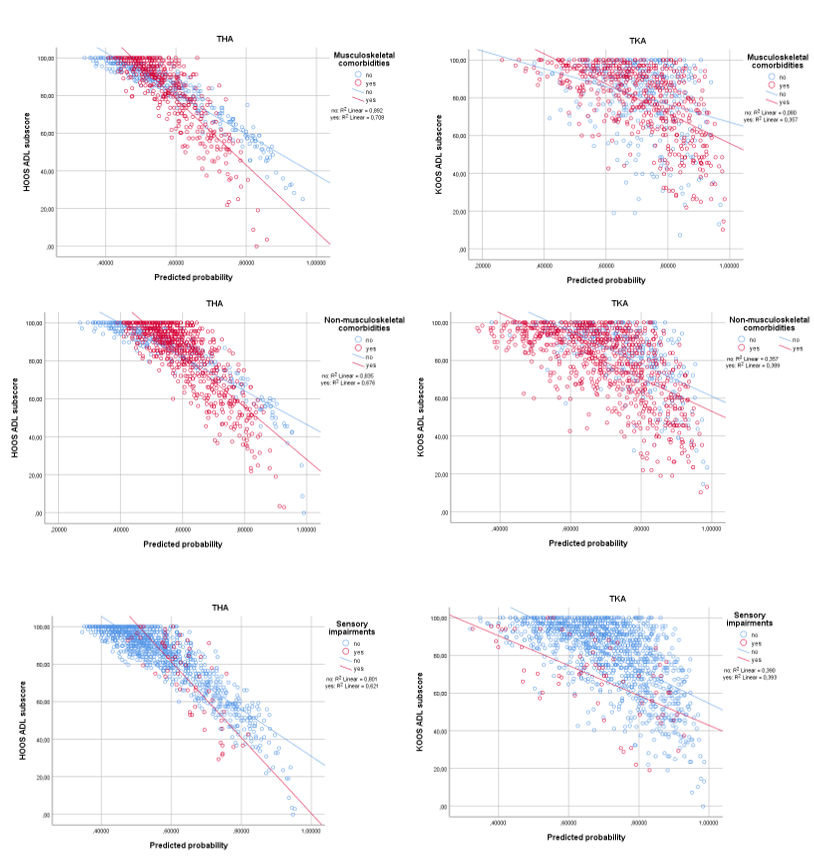


**SUPPLEMENTARY FIGURES 1-A through 2-F**. Graphs displaying the adjusted associations between recovery as measured by the ADL subscore and the predicted value of long duration of postoperative PT in THA en TKA patients. The adjusted associations are displayed stratified for the presence of a comborbidity.

Legend to supplementary figure 1

**Panels A-C-E show the associations in THA patients and panels B-D-F in in TKA patiens.**

Panel A: THA patients with or whitout a musculoskelatal comorbidity.

Panel B: TKA patients with or whitout a musculoskelatal comorbidity

Panel C: THA patients with or whitout a non- musculoskelatal comorbidity.

Panel D: TKA patients with or whitout a non- musculoskelatal comorbidity.

Panel E: THA patients with or whitout a sensory impairment.

Panel F: TKA patients with or whitout a sensory impairment.

ADL= Activities of daily living; HOOS= Hip disability and Osteoarthritis Outcome Score; KOOS= Knee injury and Osteoarthritis Outcome Score; THA=total hip arthroplasty; TKA= total knee arthroplasty.
